# History of Medicine

**Published:** 1855-12

**Authors:** 


Art. X.—*History of Medicine.* By P. V. Renouard, M. D., etc.

Here is a neat, tastefully executed volume, translated by a Cincinnati physician, and published by a Cincinnati house. The publishers have done us the favor to send us a copy of the work. Prof. Comegys has entitled himself to the thanks of his brethren by presenting this admirable history of medicine to them in so pleasing an English dress.

